# Comorbidity burden and treatment patterns in Tourette syndrome and persistent motor or vocal tic disorder

**DOI:** 10.1007/s00431-026-07009-y

**Published:** 2026-05-04

**Authors:** Attilio Sica, Benedetta Puglisi, Silja Pintar, Paolo Dalena, Egidio Barbi, Aldo Skabar

**Affiliations:** 1 University of Trieste, Trieste, Italy; 2 Institute for Maternal and Child Health - IRCCS Burlo Garofolo, Trieste, Italy

**Keywords:** Tourette syndrome, Persistent motor or vocal tic disorder, Tic spectrum disorder, Pediatrics, Attention-deficit/hyperactivity disorder

## Abstract

This study compares Persistent Motor or Vocal Tic Disorder (PMVT) and Tourette Syndrome (TS) in a pediatric population, clarifying their relationship and exploring the validity of a continuous "Tic Spectrum Disorder" (TSD). We retrospectively analyzed data from 207 pediatric patients diagnosed with PMVT or TS. Analyses included group comparisons and multivariate ordinal logistic regression to identify factors independently associated with treatment burden. The cohort comprised 128 (61.8%) TS patients and 79 (38.2%) PMVT patients. The median age at tic onset was 7 years (interquartile range 6–9). Comorbidities were more frequent in TS than PMVT (*p* < 0.001). Specifically, ADHD, Obsessive–Compulsive Disorder, Specific Learning Disorders, Oppositional Defiant Disorder, Specific Phobias (all *p* < 0.001), and Intellectual Disability (*p* = 0.046) were all more prevalent in the TS group. TS patients required more therapeutic interventions compared to PMVT (*p* < 0.001). Notably, in multivariate analysis, the treatment burden was strongly associated with the cumulative comorbidity load, although a TS diagnosis also emerged as a significant independent factor. Within the TS group, patients with ADHD showed earlier tic onset (*p* = 0.021), higher comorbidity load (*p* < 0.001), and greater therapeutic needs (*p* < 0.001).

*Conclusion*: Our study identifies the cumulative burden of comorbidities as the primary factor strongly associated with treatment requirements, with the specific tic diagnosis also contributing independently to the therapeutic burden. While these findings support the conceptualization of a continuous TSD, they shift the clinical focus toward comorbidity load as a major factor linked to healthcare demand.

**Table:** 

**What is Known:** • *Current classification distinguishes Tourette Syndrome (TS) and Persistent Motor or Vocal Tic Disorder (PMVT) based on tic types and duration, but this distinction often fails to predict therapeutic burden.* • *The existence of a continuous "Tic Spectrum Disorder" comprising both conditions is hypothesized but clinically debated.*
**What is New:** • *The cumulative comorbidity load is strongly associated with patients’ treatment requirements, although a TS diagnosis also independently contributes to the overall therapeutic burden.* • *This study expands upon recent pediatric comparisons of TS and PMVT by incorporating a broader, previously under-investigated range of neurodevelopmental comorbidities.*

**Supplementary Information:**

The online version contains supplementary material available at 10.1007/s00431-026-07009-y.

## Introduction

Although current diagnostic manuals define distinct categories of chronic tic disorders based solely on the coexistence of motor and vocal tics [1], where Tourette Syndrome (TS) requires both, while Persistent Motor or Vocal Tic Disorders (PMVT) involves only one category, emerging clinical and genomic evidence suggests these conditions do not essentially differ, but rather exist on a shared biological and clinical continuum [2–7]. Recent literature advocates for conceptualizing this spectrum as a unified “Tic Spectrum Disorder” (TSD), challenging the clinical utility of rigid categorical boundaries.

While conceptualized as a continuous spectrum, TSD clinical expression is highly heterogeneous. Demographic profiles show a well-documented male predominance and a typical tic onset between 4 and 7 years of age. While literature suggests that more clinically complex phenotypes, such as TS, manifest earlier than PMVT [2, 5], the exact interplay between these demographic factors, age at onset, specific tic diagnosis, and the overarching neurodevelopmental burden remains to be fully elucidated.

A second, highly impactful dimension of this clinical heterogeneity is the cumulative comorbidity load. Approximately 85% of TS patients have at least one psychiatric comorbidity [8], but while previous research has predominantly focused on frequent co-occurring conditions like Attention-Deficit/Hyperactivity Disorder (ADHD; 30–80%) and Obsessive–Compulsive Disorder (OCD; 28–63%) [9, 10], other relevant neurodevelopmental conditions, such as Oppositional Defiant Disorder (ODD), Autism Spectrum Disorder (ASD), Specific Learning Disorders (SLD), Intellectual Disability (ID), and Specific Phobias (SP), have been often overlooked in comparative pediatric cohorts. Consequently, it remains unclear to what extent specific tic diagnosis (PMVT vs. TS) correlates with the cumulative comorbidity load, and the resulting therapeutic needs.

To address these knowledge gaps, we compared PMVT and TS in an exclusively pediatric cohort. Recognizing ADHD’s dominant role as the earliest and most prevalent comorbidity [8, 11], we aimed to evaluate its specific impact on overall treatment demand. We hypothesized that the cumulative neurodevelopmental burden, rather than the specific tic diagnosis, is strongly associated with therapeutic needs. This analysis aims to test this hypothesis, ultimately validating current spectrum conceptualizations and informing tailored clinical stratification.

## Materials and methods

This retrospective study was conducted by reviewing medical records of pediatric patients diagnosed with primary tic disorders (PMVT and TS) at the Child Neuropsychiatry Unit of the I.R.C.C.S. Burlo Garofolo, a tertiary referral center for neurodevelopmental conditions.

### Study population

 We consecutively included all eligible patients born between 2006 and 2023, evaluated between January 2006 and December 2024 with a confirmed PMVT or TS diagnosis. No convenience sampling was used. Exclusion criteria (Supplementary Fig. 1) were:

- Incomplete clinical data (e.g. lack of essential diagnostic or follow-up information);
- Tics due to medication side effects (e.g., lithium);
- Cases where tics or tic-like movements were part of broader conditions, including neurodegenerative disorders, metabolic conditions, defined genetic syndromes, and structural brain lesions.

### Patients’ evaluation

 All patients underwent comprehensive longitudinal evaluation by child neuropsychiatrists and psychologists. Comorbidities were diagnosed according to DSM-5 criteria and the K-SADS-PL questionnaire [12] was used for psychiatric screening. When clinically indicated, standardized neuropsychological assessments supported specific diagnoses (e.g., ADHD, SLD, ID). Only formally documented diagnoses were included.

### Data collection

 Data were retrospectively extracted based on a longitudinal review of the patient's clinical history using a standardized collection form. Recorded variables included: demographics (sex, age at tic onset); tic characteristics (motor/vocal tics, PMVT/TS diagnosis); presence and number of comorbidities, including ADHD, OCD, ASD, ODD, SLD, SP, and ID; received treatment modalities and cumulative “number of treatment modalities”, defined as the count of distinct therapeutic modalities (pharmacological, psychological, or formal individualized educational plans) received throughout follow-up, regardless of duration or intensity. The TS cohort was stratified by ADHD status to determine the specific contribution of this condition to overall treatment demand. Cases with missing data were excluded (listwise deletion), and all data were anonymized.

### Statistical analysis

 The normality of continuous variables (age at tic onset, number of comorbidities, number of treatment modalities) was assessed using the Shapiro–Wilk test; since all three variables deviated significantly from normality (all *p* < 0.001), they are reported as median and interquartile range (IQR). Categorical variables are reported as absolute and percentage frequencies. Between-group comparisons for continuous variables were performed using the Wilcoxon-Mann–Whitney test. The association between number of comorbidities and number of treatment modalities was quantified using Spearman’s rank correlation coefficient. To identify factors independently associated with treatment burden, a multivariate ordinal logistic regression model was fitted with number of treatment modalities (0–3) as the ordered outcome and number of comorbidities, sex, age at tic onset, ADHD, and Tourette diagnosis as independent variables; Poisson and zero-inflated Poisson models were also explored for comparison. Model selection was based on Akaike Information Criterion (AIC). Variance Inflation Factors (VIFs) were calculated to assess multicollinearity. Statistical significance was set at *p* < 0.05. All analyses were performed in R (version 4.5.2).

## Results

### Baseline characteristics of the study population

**Fig. 1:**
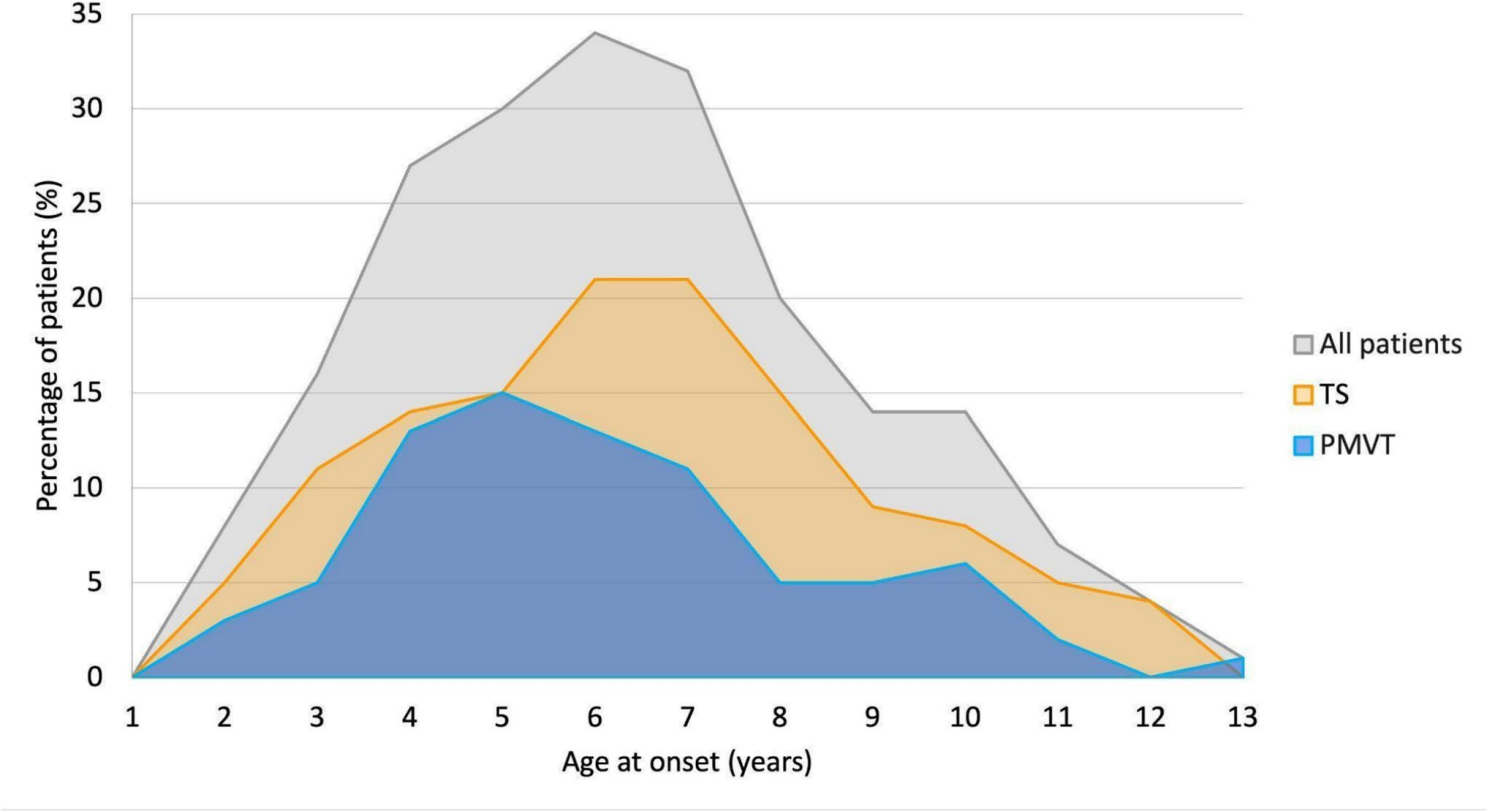
Age distribution at onset in patients with PMVT and TS. The total sample (All patients) is also represented. PMVT: Persistent Motor or Vocal Tic Disorder; TS: Tourette Syndrome

 Medical records of 236 patients were assessed for eligibility (Supplementary Fig. 1). Twenty-nine patients were excluded due to insufficient clinical documentation (*n* = 27), tics related to medication side effects (*n* = 1), or tics occurring within complex neurological syndromes (*n* = 1), yielding a final sample of 207 participants. The cohort comprised 157 males (75.8%) and 50 females (24.2%) (male-to-female ratio ≈ 3:1) (Table 1). Sex distribution was similar in PMVT (70.9% male, 29.1% female) and TS (78.1% male, 21.9% female). Regarding the diagnostic distribution, among the 207 patients included in the study, 128 (61.8%) fulfilled the diagnostic criteria for TS. The remaining 79 patients (38.2%) were diagnosed with PMVT: specifically, 73 (35.3%) presented with persistent motor tics only, whereas 6 (2.9%) presented with persistent vocal tics only. Median age at tic onset (Fig. 1) for the entire cohort was 7 years (IQR 6–9). Age at tic onset was also comparable between PMVT and TS, with no statistically significant difference observed (median 7, IQR 5–8 vs. median 7, IQR 6–9; *p* = 0.224).

**Table 1:** Baseline demographics, clinical characteristics, and treatment data of the study population in the total cohort and stratified by diagnostic group. Data are presented as number (%) for categorical variables, and as median and interquartile range (IQR) for continuous variables. *P*-values indicate the statistical significance of the comparisons between the PMVT and TS groups. Statistically significant *p*-values (< 0.05) are shown in bold. ADHD: Attention-Deficit/Hyperactivity Disorder; ASD: Autism Spectrum Disorder; ID: Intellectual Disability; IQR: Interquartile Range; OCD: Obsessive–Compulsive Disorder; ODD: Oppositional Defiant Disorder; PMVT: Persistent Motor or Vocal Tic Disorder; SD: Standard Deviation; SLD: Specific Learning Disorder; SP: Specific Phobias; TS: Tourette Syndrome

Variable	Total Cohort (*n* = 207)	PMVT (*n* = 79)	TS (*n* = 128)	*p*-value
Sex, No. (%)				0.313
Male	157 (75.8)	56 (70.9)	100 (78.1)	
Female	50 (24.2)	23 (29.1)	28 (21.9)	
Age at tic onset (years)				0.224
Median (IQR)	7 (6–9)	7 (5–8)	7 (6–9)	
Number of comorbidities				** < 0.001**
Median (IQR)	0 (0–2)	0 (0–0)	1 (0–3)	
Specific comorbidities, No. (%)				
ADHD	50 (24.2)	4 (5.1)	46 (35.9)	** < 0.001**
ASD	6 (2.9)	2 (2.5)	4 (3.1)	1.000
ID	7 (3.4)	0 (0.0)	7 (5.5)	**0.046**
OCD	46 (22.2)	4 (5.1)	42 (32.8)	** < 0.001**
ODD	50 (24.2)	4 (5.1)	46 (35.9)	** < 0.001**
SLD	25 (12.1)	1 (1.3)	24 (18.8)	** < 0.001**
SP	41 (19.8)	4 (5.1)	37 (28.9)	** < 0.001**
Number of treatment modalities				** < 0.001**
Median (IQR)	0 (0–2)	0 (0–0)	1 (0–3)	
Specific treatments, No. (%)				
Psychotherapy	71 (34.3)	14 (17.7)	57 (44.5)	** < 0.001**
Pharmacological therapy	55 (26.6)	5 (6.3)	50 (39.1)	** < 0.001**
Educational support	45 (21.7)	4 (5.1)	41 (32.0)	** < 0.001**

### Comorbidities

**Fig. 2:**
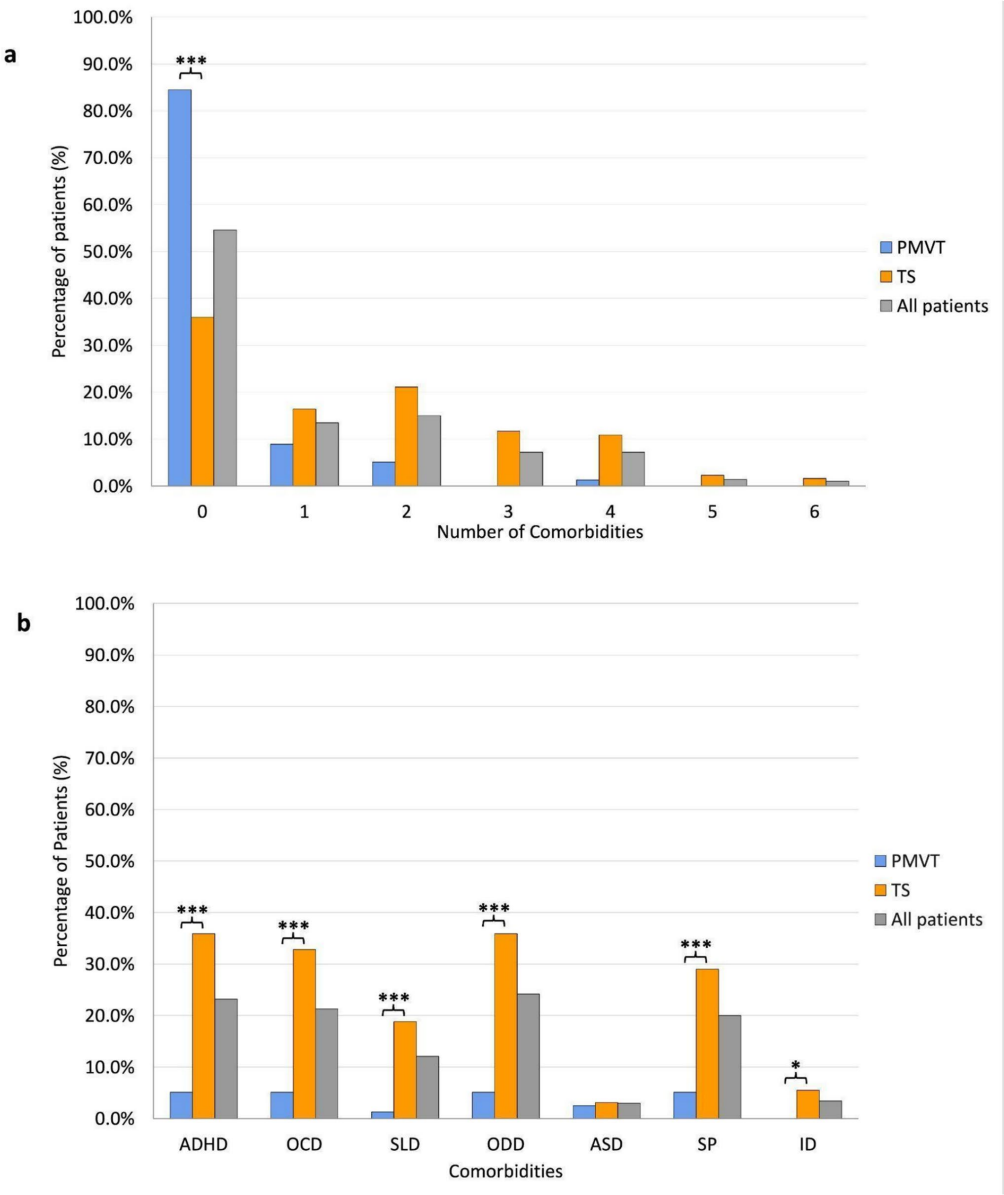
Comorbidity profiles in PMVT and TS: TS is associated with a greater occurrence of multiple comorbidities, while the absence of comorbidities is more frequent in PMVT. **a**: Bar charts illustrating the distribution of the number of comorbidities (0 to 6) in patients with PMVT, TS, and in the total sample (All patients). **b**: frequency of individual psychiatric and neurodevelopmental comorbidities across the same groups. (*p < 0.05; ***p < 0.001). ADHD: Attention-Deficit/Hyperactivity Disorder; ASD: Autism Spectrum Disorder; PMVT: Persistent Motor or Vocal Tic Disorder; ID: Intellectual Disability; OCD: Obsessive–Compulsive Disorder; ODD: Oppositional Defiant Disorder; SLD: Specific Learning Disorder; SP: Specific Phobia; TS: Tourette Syndrome

 Regarding comorbidities (Fig. 2, Table 1), 93 patients (45.4%) presented at least one associated condition. The presence of at least one comorbidity was significantly more common in patients with TS (82 patients, 64.1%) than in those with PMVT (12 patients, 15.2%; *p* < 0.001). The number of comorbidities was significantly higher in TS compared with PMVT (median 1, IQR 0–3 vs. median 0, IQR 0–0; *p* < 0.001). Notably, none of the six patients with isolated chronic vocal tic disorder had comorbidities. When individual comorbidities were analyzed, ADHD (TS: 35.9% vs. PMVT: 5.1%), OCD (32.8% vs. 5.1%), SLD (18.8% vs. 1.3%), ODD (35.9% vs. 5.1%), and SP (28.9% vs. 5.1%) were all significantly more frequent in TS than in PMVT (*p* < 0.001 for each). ID was identified in 5.5% of TS and was absent in PMVT, and this difference was statistically significant (*p* = 0.046). ASD was rare and showed no significant difference between the two groups (TS 3.1% vs. PMVT 2.5%; *p* = 1.00).

**Fig. 3:**
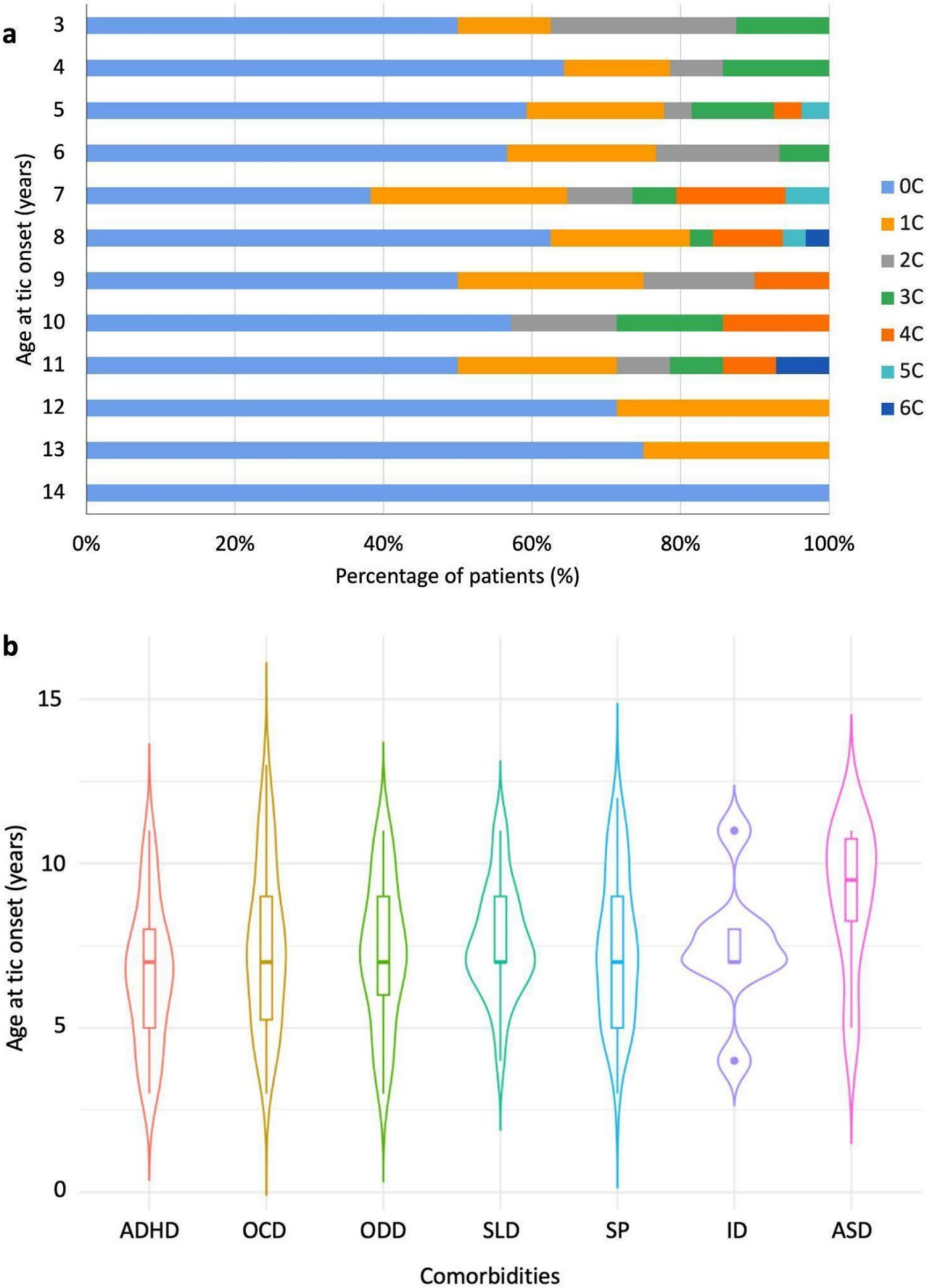
Relationship between age at tic onset and comorbidity profile: earlier onset is associated with ADHD and SP, while ASD shows a trend toward later onset. **a**: stacked bar chart showing the distribution of the number of comorbidities (0C to 6 C) according to age at tic onset. **b**: violin plots depicting the age distribution at tic onset across individual comorbidities. ADHD: Attention-Deficit/Hyperactivity Disorder; ASD: Autism Spectrum Disorder; ID: Intellectual Disability; OCD: Obsessive–Compulsive Disorder; ODD: Oppositional Defiant Disorder; SLD: Specific Learning Disorder; SP: Specific Phobia

We analyzed the influence of specific comorbidities on the age at tic onset (Fig. 3). Age at tic onset was significantly associated with ADHD: patients with ADHD had an earlier onset compared to those without (median 7, IQR 5–8 vs. median 7, IQR 6–9; *p* = 0.021). ASD showed a trend toward later onset (median 9.5, IQR 8.25–10.75 vs. median 7, IQR 6–9; *p* = 0.077), which did not reach statistical significance, consistent with the small number of ASD cases (*n* = 6). No significant variations in the age at tic onset were observed for other comorbidities (ID: *p* = 0.821; OCD: *p* = 0.704; ODD: *p* = 0.949; SLD: *p* = 0.374; SP: *p* = 0.776).

### Treatment

**Fig. 4:**
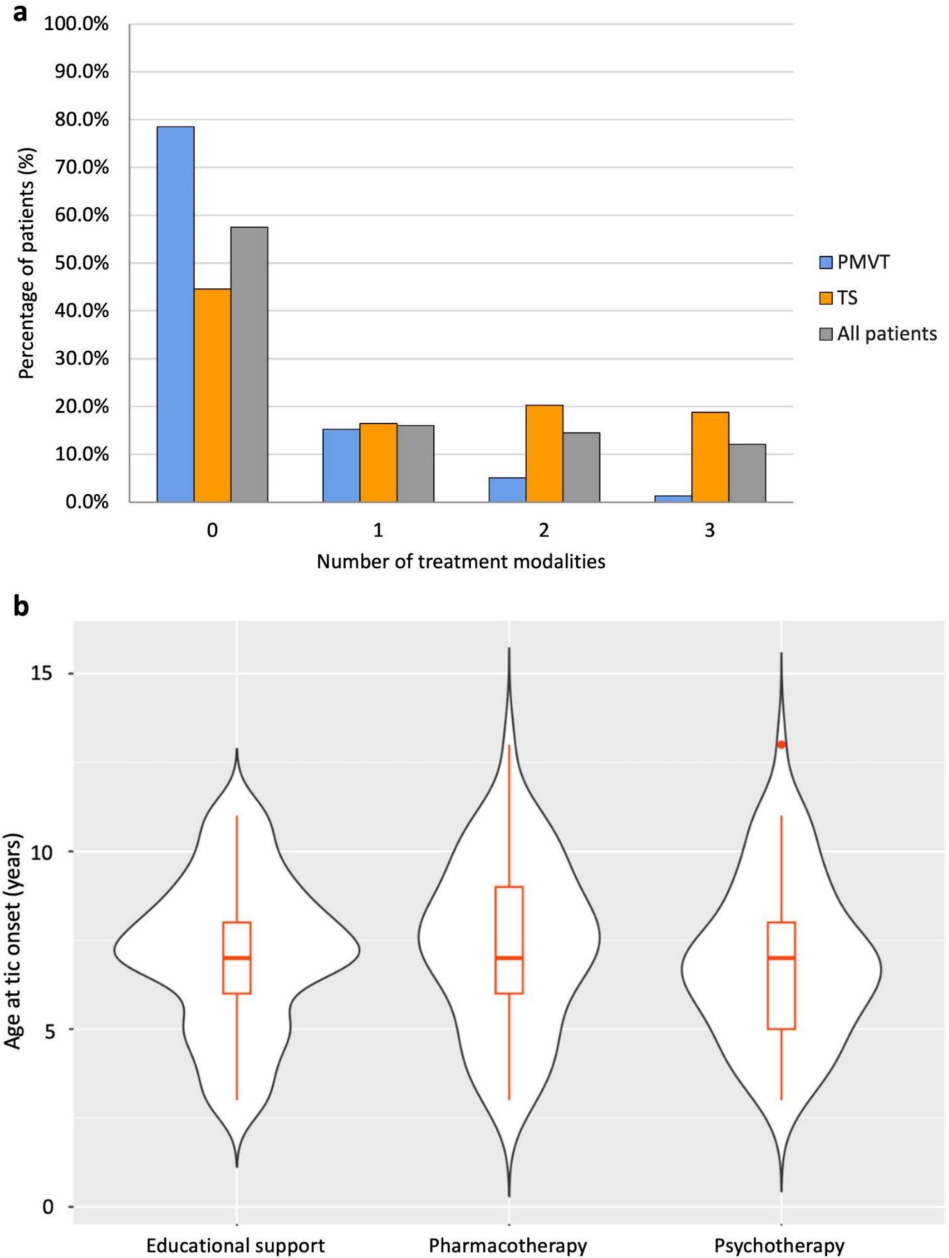
Distribution of treatment modalities in patients with PMVT and TS. **a**: bar chart illustrating the distribution of the number of treatments (defined as the count of distinct therapeutic modalities; 0 to 3) received by patients with PMVT, TS, and in the total sample (All patients). **b**: violin plots depicting the age at tic onset in relation to specific treatment modalities (educational support, pharmacotherapy, and psychotherapy). PMVT: Persistent Motor or Vocal Tic Disorder; TS: Tourette Syndrome

 Treatment patterns differed substantially between groups (Fig. 4). In the whole cohort, 119 patients (57.5%) required no treatment, 33 (15.9%) received one type of treatment, 30 (14.5%) received two types of treatments, and 25 (12.1%) received all three modalities. Among PMVT patients, 62 (78.5%) received no treatment, 12 (15.2%) received one, 4 (5.1%) received two, and only 1 (1.2%) required three. In contrast, TS patients were more likely to receive multiple interventions: 57 (44.5%) received no treatment, 21 (16.4%) received one type, 26 (20.3%) received two types, and 24 (18.8%) received three. The number of treatment modalities was significantly higher in TS than in PMVT (median 1, IQR 0–2 vs. median 0, IQR 0–0; *p* < 0.001). All three types of therapeutic interventions were significantly more frequent in the TS group: psychotherapy (TS: 44.5% vs. PMVT: 17.7%, *p* < 0.001), pharmacological therapy (39.1% vs. 6.3%, *p* < 0.001), and educational support (32.0% vs. 5.1%, *p* < 0.001).

We analyzed whether the age at tic onset influenced the therapeutic strategy (Fig. 4). We found no significant differences in the average age at tic onset between patients receiving pharmacotherapy and those who did not (median 7, IQR 6–9 vs. median 7, IQR 5–9; *p* = 0.694). Similarly, no significant association was found regarding the allocation of educational support (median 7, IQR 6–8 vs. median 7, IQR 6–9; *p* = 0.741). Patients receiving psychotherapy showed a trend toward an earlier age at tic onset compared to those not receiving it, but this difference did not reach statistical significance (median 7, IQR 5–8 vs. median 7, IQR 6–9; *p* = 0.088).

**Fig. 5:**
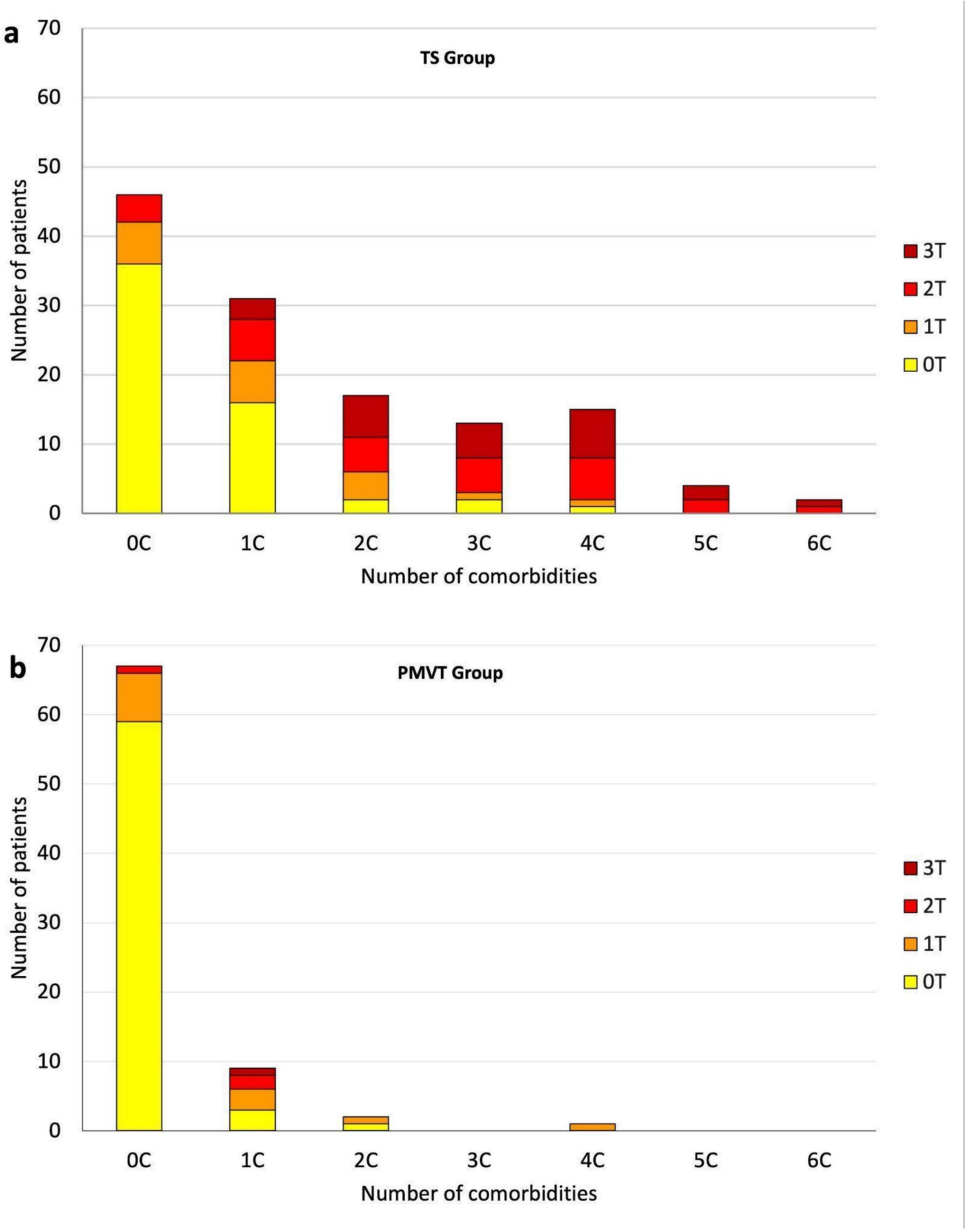
Relationship between the number of comorbidities and treatment requirements. **a**: distribution of patients in the TS group. **b**: distribution of patients in the PMVT group. The color code represents the number of treatments (defined as the count of distinct therapeutic modalities; 0 T to 3 T) required for each level of comorbidity (0C to 6 C). PMVT: Persistent Motor or Vocal Tic Disorder; TS: Tourette Syndrome

A strong positive correlation was found between the total number of comorbidities and the number of therapeutic interventions required (Spearman’s rank correlation coefficient = 0.70, *p* < 0.001) (Fig. 5): 95% of patients without comorbidities required no treatment, whereas those with ≥ 3 comorbidities frequently needed ≥ 2 interventions; all patients with 5–6 comorbidities required multiple treatments.

In multivariate ordinal logistic regression, the number of comorbidities was the factor most strongly associated with the number of treatment modalities (aOR = 2.48 per additional comorbidity, *p* < 0.001). TS diagnosis was also a significant independent factor (aOR = 2.07, *p* = 0.048). Sex, age at tic onset, and ADHD were not significant in the multivariable model (all p > 0.05). VIF values for all covariates ranged from 1.03 to 2.26, confirming the absence of multicollinearity. The ordinal model showed the best fit among the models explored (AIC = 379.5), outperforming Poisson regression (AIC = 430.8); a zero-inflated Poisson model showed numerical instability and was therefore excluded.

### TS patients with and without ADHD

 Among the 128 TS patients, 46 (35.9%) also had ADHD, while 82 (64.1%) did not. The ADHD subgroup had a significantly earlier age at tic onset (median 7, IQR 5–8 vs. median 8, IQR 6–9; *p* = 0.006), a significantly higher number of comorbidities (median 3, IQR 2–4 vs. median 0, IQR 0–1; *p* < 0.001), and received significantly more treatment modalities (median 2, IQR 1–3 vs. median 0, IQR 0–1; *p* < 0.001). In terms of individual comorbidities, ODD was significantly more prevalent in TS + ADHD than in TS without ADHD (69.6% vs. 17.1%, *p* < 0.001), as were SLD (32.6% vs. 11.0%, *p* = 0.004) and SP (41.3% vs. 22.0%, *p* = 0.026). No significant differences were observed between the two subgroups for OCD (37.0% vs. 30.5%; *p* = 0.557), ASD (4.3% vs. 2.4%; *p* = 0.618), and ID (10.9% vs. 2.4%; *p* = 0.097). In terms of individual treatment modalities, TS + ADHD patients were significantly more likely to receive pharmacological therapy (65.2% vs. 24.4%, *p* < 0.001), psychotherapy (73.9% vs. 28.0%, *p* < 0.001), and educational support (63.0% vs. 14.6%, *p* < 0.001).

## Discussion

This study compares TS and PMVT within an exclusively pediatric population. While previous research has explored this comparison [2, 3, 5], our work provides new insights by focusing on children and adolescents (age at tic onset 3–14 years) and by including comorbidities not previously considered, such as ASD, SLD, ID, and SP.

Consistent with prior reports [13], in our cohort TS was more frequent than PMVT (128 vs 79 cases); however, PMVT is probably underdiagnosed, because of milder symptoms and lower referral rates. Our demographic data confirm a substantial overlap between TS and PMVT in terms of male-to-female ratio (≈ 3:1) [14, 15]. This male predominance, common to many neurodevelopmental conditions, likely reflects a complex interplay of both biological and environmental factors. For instance, it might be partially influenced by referral biases, as females often present with more internalizing comorbidities and less prominent tic manifestation, delaying detection [16].

Age at tic onset was similar between groups (around 7 years). This diverges from the majority of the literature, including the recent pediatric study by Nilles et al. (2025) [5], which reported a significantly later onset for PMVT compared to TS. This discrepancy may be primarily methodological: given the inherent variability in retrospectively establishing the exact age at tic onset from parental recall, our sample size might lack the statistical power to detect the relatively small temporal differences visible in larger registries. Furthermore, criteria used across different clinical settings to define the exact onset of persistent tics versus transient manifestations may be heterogeneous and contribute to these diverging results.

Collectively, the substantial demographic overlap between TS and PMVT found in our study aligns with prior research suggesting a shared etiology across the TSD. Alongside genetics [4], the existence of common environmental contributions, ranging from psychosocial moderators of well-being [17] to potential biological triggers [18], is increasingly scrutinized. Furthermore, the occurrence of the same comorbidities in both TS and PMVT, albeit with different frequencies, alongside similar clinical management requirements, further underscores this clinical continuum and supports a unified therapeutic framework.

Despite this overlap, clinical expression varies significantly, particularly regarding the comorbidity burden. Overall, we observed a lower comorbidity prevalence than previously reported: while prior literature indicates that over 80% of patients with TS present with at least one comorbid disorder [13], in our TS cohort this percentage was lower (64.1%). This might be explained by our cohort’s pediatric nature, as our patients may not yet have developed late-onset conditions like OCD, and by the fact that we did not investigate mood or anxiety disorders.

Importantly, while univariate comparisons showed that TS patients exhibited significantly higher comorbidity rates and therapeutic requirements than PMVT patients, our multivariate regression model clarified the specific factors associated with this need. We found that the treatment burden is primarily associated with the cumulative comorbidity load. As could be expected, TS diagnosis also emerged as a significant independent factor, suggesting it carries additional treatment needs beyond those explained by comorbidity load alone. In contrast, after adjusting for the total comorbidity count, variables such as age at tic onset, sex, and ADHD presence failed to retain statistical significance as predictors of the number of received treatment modalities. Thus, while the specific tic diagnosis remains clinically relevant, therapeutic decision-making is heavily influenced by the cumulative burden of co-occurring conditions.

This underscores a critical limitation in current diagnostic classifications, which only rely on tic characteristics without considering comorbidities’ role in determining care requirements. Consequently, clinical management should always consider the full spectrum of associated neurodevelopmental conditions, as these may impact quality of life more than tics themselves [19]. From this perspective, we support the adoption of the concept of TSD.

Within this spectrum, while TS generally represents a more complex phenotype than PMVT, TS with co-occurring ADHD emerges as the most severe profile: ADHD + TS patients presented with earlier onset and increased demand for both medical and educational interventions, consistent with the multimodal treatment strategies advocated by current European guidelines [19]. The known association of ADHD with ODD and SLD [20] was also confirmed. Regarding the specific role of ADHD, in our multivariate analysis it was not independently associated with treatment burden when adjusting for the total comorbidity count (VIF = 2.17, indicating no severe multicollinearity). However, this result should be interpreted with caution, since in a pragmatic clinical perspective ADHD remains a major contributor to care requirements, possibly due to its high prevalence and substantial impact on overall quality of life. As a matter of fact, its cumulative load and its impact on therapeutic needs should be interpreted within the context of the complex neurodevelopmental phenotype in which it is frequently embedded.

Within a TSD framework, our results support the need for routine screening of psychiatric and neurodevelopmental comorbidities in all children with chronic tics, regardless of their specific diagnosis. Although a specific TS or a PMVT diagnosis retains independent clinical relevance, relying on it exclusively risks underestimating the profound impact of the overall neurodevelopmental burden. Consequently, early identification of co-occurring conditions is essential to refine risk stratification and to guide more personalized and proactive management strategies.

Our study has several limitations. The retrospective, single-center design limits generalizability. Without validated measures of tic severity, such as the Yale Global Tic Severity Scale, or standardized assessment tools for individual comorbidities, our evaluation of clinical burden was restricted to assessing “therapeutic burden” (inferred from the number of treatment modalities) rather than “tic severity” (not assessed). Although integrating pharmacological, psychological, and educational interventions into a single metric (“number of treatment modalities”) may overshadow their heterogeneous indications and accessibility, this approach was specifically chosen to capture the complex, multidimensional management typically required by this patient population. Furthermore, we must acknowledge the possibility of residual confounding, as unmeasured factors, such as socioeconomic status or clinical referral pathways, might have influenced the observed associations between comorbidity load and treatment requirements. Moreover, the retrospective data collection prevented us from distinguishing tic-specific medications from those prescribed for comorbidities. Additionally, our data did not encompass mood and anxiety disorders, and our sample size limited the statistical power to thoroughly investigate uncommon comorbidities and to adequately assess demographic interactions. Finally, to capture the maximal cumulative clinical burden, data were extracted across the patients' entire longitudinal follow-up, preventing the use of a standardized cross-sectional “age at evaluation”. Consequently, this approach precluded the analysis of age-dependent comorbidity trajectories or prospective diagnostic evolution (e.g., conversion from PMVT to TS).

Future multicenter, prospective studies with larger samples should further investigate demographic differences and further explore uncommon comorbidities. Specifically, the identification of seven ID cases in our TS group, rarely described in association with tic disorders, suggests broader phenotypes that deserve further exploration. Long-term follow-up of provisional tics is also needed, as emerging evidence [21] suggests they may represent an early TSD stage rather than a transient condition. Finally, prospective longitudinal studies are needed to monitor the evolution from PMVT to TS, accounting for typical developmental delays in vocal tic onset [8].

In conclusion, our results support conceptualizing primary tic disorders as a continuous spectrum (TSD), with TS representing the more complex end, characterized by higher comorbidity rates and greater therapeutic needs. By demonstrating that treatment requirements are strongly associated with the cumulative neurodevelopmental comorbidity burden, while also recognizing the independent contribution of a specific TS diagnosis, our findings add critical evidence to the literature. Recognizing TSD as a diagnostic entity would enhance our ability to capture this heterogeneity and improve clinical outcomes through personalized, comorbidity-centered, needs-based interventions.

## Supplementary Information

Below is the link to the electronic supplementary material.

Supplementary file1 (PDF 183 KB)

## Data Availability

The data that support the findings of this study are available from the corresponding author upon reasonable request.

## Abbreviations

ADHD: Attention-Deficit/Hyperactivity Disorder
aOR: Adjusted Odds Ratio
ASD: Autism Spectrum Disorder
PMVT: Persistent Motor or Vocal Tic Disorder
DSM-5: Diagnostic and Statistical Manual of Mental Disorders, Fifth Edition
ID: Intellectual Disability
IQR: Interquartile Range
OCD: Obsessive-Compulsive Disorder
ODD: Oppositional Defiant Disorder
SLD: Specific Learning Disorders
SP: Specific Phobias
TS: Tourette Syndrome
TSD: Tic Spectrum Disorder
VIF: Variance Inflation Factor
