# Supplementary material for: Comorbidity burden and treatment patterns in Tourette syndrome and persistent motor or vocal tic disorder
Source: Eur J Pediatr. 2026 May 4;185(5):343. doi: 10.1007/s00431-026-07009-y (PMC13139208; doi:10.1007/s00431-026-07009-y)
Supplement: Supplementary file 1 — Supplementary file1 (PDF 183 KB) [file 431_2026_7009_MOESM1_ESM.pdf]

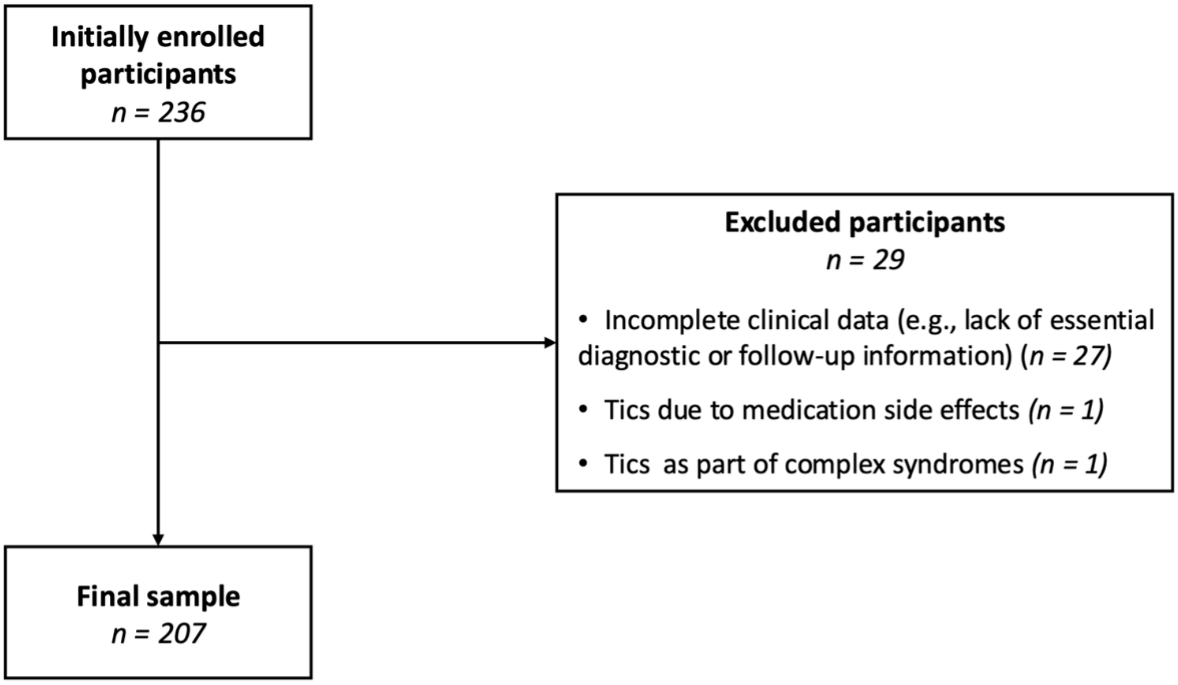

Supplementary Figure 1. Flowchart of participant selection. Of the 236 medical records assessed for eligibility, 29 patients were excluded based on predefined criteria, resulting in a final sample of 207 participants
